# The Emergence of Educational Hypogamy in India

**DOI:** 10.1007/s13524-020-00888-2

**Published:** 2020-06-10

**Authors:** Zhiyong Lin, Sonalde Desai, Feinian Chen

**Affiliations:** 1 Department of Sociology, University of Maryland, 2112 Art-Sociology Building, College Park, MD 20742 USA; 2 Maryland Population Research Center, University of Maryland, College Park, MD 20742 USA

**Keywords:** Assortative mating, Gender, Education, India

## Abstract

With rising education among women across the world, educational hypergamy (women marrying men with higher education) has decreased over the last few decades in both developed and developing countries. Although a decrease in hypergamy is often accompanied by increasing homogamy (women marrying men with equal levels of education), our analyses for India based on a nationally representative survey of India (the India Human Development Survey), document a considerable rise in hypogamy (women marrying partners with lower education) during the past four decades. Log-linear analyses further reveal that declining hypergamy is largely generated by the rise in education levels, whereas hypogamous marriages continue to increase even after marginal distributions are taken into account. Further multivariate analyses show that highly educated women tend to marry men with lower education but from more privileged families. Moreover, consanguineous marriages, which exemplify strong cultural constraints on spousal selection in certain parts of India, are more likely to be hypogamous than marriages not related by blood. We argue that the rise in hypogamous marriage by education paradoxically reflects deep-rooted gender scripts in India given that other salient social boundaries are much more difficult to cross.

## Introduction

Demographers have long been interested in assortative mating because it involves the maintenance and destruction of social boundaries, and it is often indicative of rigidity in social norms as well as social mobility in general (see review by Schwartz 2013). Previous studies have consistently shown that as societies develop, achieved traits (e.g., education and occupation) become increasingly important criteria of mate selection, whereas the roles of ascribed traits (e.g., race/ethnicity and religion) weaken (Blossfeld 2009; Kalmijn and Flap 2001; Qian and Lichter 2007). One key factor responsible for this change is the impressive expansion in education, especially the increase in educational opportunities and attainment for women around the world. As such, changes and variations in educational assortative mating are particularly interesting in societies undergoing a dramatic socioeconomic transformation. Following an increase in women’s education all over the world, educational hypergamy (women marrying men with higher education) has been decreasing over the last few decades in both developed and developing countries; this trend, often referred to as “the end of hypergamy,” is considered to have broad implications for family dynamics and gender equality (Esteve et al. 2012, 2016; Van Bavel et al. 2018).

Recent studies on educational assortative mating have shown that nearly 90% of the variation in educational hypergamy can be accounted for by gender inequality in education (Esteve et al. 2012). Transformation of marriage patterns from male advantage to partner equality to female advantage can occur without any changes in the underlying gender stratification system and can be explained simply by general expansion in education opportunities benefitting both men and women (Esteve et al. 2016). At the same time, it is undeniable that in many developed countries, marriage as an institution has changed profoundly from one that upholds the breadwinner-homemaker ideal to one that is increasingly gender-egalitarian (Cherlin 2004). The rise of homogamy or hypogamy is thus concomitant with such a trend.

Trends in educational assortative mating in India conform to the global experience in some ways but deviate from it in others. Educational attainment for both men and women has sharply increased in recent years, and the gap between men and women has decreased substantially. As expected, educational hypergamy has declined accordingly. At the same time, women still lag behind men considerably in education, and the gap is far from disappearing. For example, based on a recent report by the government of India (Ministry of Human Resource Development 2018), about 40% of females aged 7 and older are illiterate, compared with only 20% for their male counterparts. Nonetheless, educational hypogamy in India has risen sharply. As shown in our work here, only about 5% of women in the 1970s marriage cohorts married men with lower education than themselves, but this figure rose to about 20% in recent marriage cohorts. Although incidences of educational hypogamy have increased globally, they are often limited to countries where a reverse gender gap in education has occurred and where gender norms and the marriage institution have typically become more relaxed. In India, neither shift has taken place. Gender inequality is pervasive, and patriarchal norms still have a stronghold in the Indian society (Chakrabarti and Biswas 2012; Kishor and Gupta 2004; Weitzman 2014). In the absence of either a disappearing/reversed gender gap in education or any dramatic shifts in gender norms, how can we reconcile the Indian experience with the global trends?

We argue that two aspects of Indian experience deserve particular attention. First, given the continued prevalence of arranged marriages in India and nearly universal marriage (Allendorf and Pandian 2016), partner pools may be more restricted for Indian women, resulting in a greater emphasis on ascribed characteristics (e.g., caste and kinship network) over achieved characteristics (e.g., education). Second, the nature of educational and employment structures in India does not reward educational attainment consistently with the assumptions underlying global assortative mating literature, and this dissonance has increased over time. Improved education does not necessarily result in higher incomes for women given that labor force participation for women seems to decline rather than increase with education in India (Chatterjee et al. 2018). Thus, rising hypogamy in India may have a lower social significance than in other countries and may be more easily accommodated within traditional marriage power dynamics.

Our study examines trends and variations in spousal educational differences using data from the India Human Development Survey (IHDS) carried out in 2011–2012. The sample consists of 28,311 women aged 15–49 representing marriage cohorts of the 1970s, 1980s, 1990s, and post-2000. Using log-linear models, we first examine whether these changes can be accounted for by the changes in the distribution of education of the wife and husband. Following that, we examine several social processes underlying increases in hypogamy by using a series of multilevel models that capture the nature of the marriage market and educational systems while controlling for the state-level educational distribution of men and women eligible for marriage. Our analyses focus on salient social boundaries, such as socioeconomic status (i.e., status exchanges between education and family socioeconomic status); India-specific phenomena, such as arranged marriages, consanguineous marriages, and other marriage practices; and the fields of education.

## Assortative Mating: Global Explanations

The simultaneous increase in women’s education and the decrease in educational hypergamy is nearly universal and well documented in the literature (Esteve et al. 2012, 2016). However, the underlying causes of this transformation remain contested. Three sets of explanations have received considerable attention: (1) changing educational distribution, (2) changing gender norms, and (3) status exchange.

### Changing Educational Distribution

The dominant theme in assortative mating literature argues that marital hypergamy is replaced by increasing homogamy in countries where gender equality in education is observed and by educational hypogamy, in which women’s education outpaces men’s. Research has documented a number of channels through which educational expansion may affect this process. As shown for China and the United States (Han 2010; Schwartz and Mare 2005), rising female education may lead to a greater earning potential, thereby increasing women’s chances of marrying men with similarly high education and earning potential. Evidence from the Netherlands and the United States has shown that expanding education provides more opportunities for women to meet their potential partners at school, which in turn increases the chance of educational similarities (Kalmijn and Flap 2001; Mare 1991).

Whether the end of hypergamy results in educational homogamy or crosses over to educational hypogamy (women marry partners with lower education) remains a matter of debate. In the United States and almost all European countries, women’s educational attainment has surpassed men’s (Van Bavel et al. 2018). This changing educational distribution may result in more women marrying men with a lower level of education simply because the partner pool is changing (De Hauw et al. 2017; Schwartz and Han 2014). This would imply that increasing hypogamy is due to changes in educational distribution but not necessarily due to any fundamental shifts in gender norms.

### Changing Gender Norms

The second set of explanations focuses on changing gender norms. Some of these explanations suggest that higher education for women may lead to greater gender egalitarianism, resulting in a higher level of gender symmetrical mating patterns in many social contexts (Schwartz 2013). Prior research has also focused on changes in the underlying rate of hypogamy that are independent of distributional changes. This research has argued that with dramatic increases in both women’s education and their labor force participation, women become less dependent on their husbands’ incomes and hence have greater freedom to choose a partner on the basis of non-economic criteria, which in turn may lead to less homogamous marriages (Smits 2003; Smits and Park 2009).

This literature assumes that growth in education for women is linked to greater economic opportunities. However, ample evidence from gender and development literature suggests that initial industrialization restricts women’s opportunities for work in traditional sectors, particularly agriculture, without creating alternative opportunities (Boserup 1970). Claudia Goldin (1995) has shown that economic development has a U-shaped relationship with women’s employment, with women being most likely to be employed in the least and most developed societies. Thus, whether rising education leads to increasing employment and changes in the balance of marital power in developing countries remain empirical questions.

### Status Exchange

Mate selection among young singles is constrained by the pool of appropriate partners (Kalmijn 1998; Qian and Lichter 2018). A focus on educational distribution in the available partner pool centers on one characteristic: education. However, the partner pool may also be affected by other characteristics of candidates in the marriage market (Schwartz 2013).

Indeed, the end of educational hypergamy as a global trend may coexist with the persistence of more traditional matching patterns on other dimensions (Van Bavel et al. 2018). The status exchange theory (Davis 1941; Merton 1941) provides a conceptual basis for understanding assortative mating along multiple dimensions. It notes that individuals compensate for the lack of one trait by offering other desirable traits to potential mates (Schwartz et al. 2016). This theory has been used widely in the study of interracial marriages, in which partners trade racial status for socioeconomic status (e.g., education) (Gullickson and Torche 2014; Hou and Myles 2013). Following the reversal of the gender gap in education in many developed countries, some recent studies have started documenting status exchange among women who marry below their educational status. Evidence from the United States has shown that women are more likely to marry below their educational status when their husbands make more money or are from more privileged families (Qian 2017; Schwartz et al. 2016). Studies on mainland China and Hong Kong also found the status exchange of education for residential status between migrants and local residents (Qian and Qian 2017; Zhou 2016).

## Challenge of Indian Experience

India provides an interesting site for studying changes in assortative mating in a society where educational attainment has risen alongside declining rather than increasing female labor force participation (Klasen and Pieters 2015). Previous research controlling for family income and a variety of sociodemographic factors has found that Indian women with moderate levels of education are less likely to work than either illiterate women or college graduates (Chatterjee et al. 2018; Klasen and Pieters 2015). Both cultural norms restricting women’s movement and structural constraints, such as a lack of appropriate job opportunities for educated women, are considered to play important roles in explaining the stagnation of female labor force participation in India. Although the low association between Indian women’s education and labor force participation is unusual, it is not unique, having also been documented among Arab, Iranian, and Korean women (Read and Cohen 2007; Read and Oselin 2008). In these contexts, women’s education may be encouraged as a cultural strategy to ensure that children receive proper socialization rather than as a path to higher earning, and thus may contribute to the reproduction of patriarchal gender relations. The lack of association between women’s increasing education and better economic prospects could contribute to the difference in educational assortative mating between Western and non-Western countries.

The marriageable pool for Indian couples is also limited by the norms that define the social boundaries within which marriage may occur. India is distinguished by its continued prevalence of arranged marriage and the narrow definition of who is considered an appropriate marriage partner. Although women are increasingly involved in the selection of their husbands, this change is modest; a substantial majority of the marriages are still arranged by family members, with self-selection accounting for less than 5% of marriages (Allendorf and Pandian 2016).

Kinship and geography also play a role in mate selection. In southern India, cousins and maternal uncles are considered preferred marriage partners. In northern India, such unions would be considered incestuous, and women are expected to marry outside of not only their own family but also their natal village (Chowdhry 2007; Desai et al. 2010). The norm of patrilocal exogamy, wherein wives migrate to coreside with their husbands’ kin outside their natal villages, is more pronounced in northern India; consanguineous marriages, which often happen within a community, are encouraged in southern India (Rammohan and Vu 2018).

In addition, caste is still the single most important criterion in the marriage market characterized by a strong preference for intracaste marriage (Banerjee et al. 2013; Goli et al. 2013). Another aspect of marriage in India is its universality; the proportion of 30- to 34-year-old women who are single barely changed between 1971 and 2011, rising from 0.9% to 3.7% (Yeung et al. 2018). Whereas Chinese women, for example, may choose to remain single rather than marry a less-educated mate (Qian and Qian 2014), this is not an option easily open to Indian women. All these normative restrictions limit the eligible pool of appropriate mates and differentiate India from the other social contexts studied in previous research on educational assortative mating.

Like other parts of the world, India has also made encouraging progress in increasing school participation, especially among women (Kingdon 2007). Although still lagging behind men, Indian women have achieved dramatic improvement in educational attainment (Rammohan and Vu 2018). The increase in women’s education has been shown to play an important role in demographic changes in India, with maternal education being positively associated with declining fertility (Bhattacharya 2006), children’s health (Vikram et al. 2012), and children’s education (Behrman et al. 2008). However, because of its strong hierarchical gender regime, women’s education may be treated differently in India than in other countries. For example, a qualitative study in southern India documented an ambivalent perception of women’s education: the education of girls holds the promise of upward mobility through marriage, but it also raises fear about producing a “spoiled bride” (Still 2011).

Given the rigid gender scripts in the Indian society (Desai and Andrist 2010), it is not surprising that some studies have suggested that the most important quality for women in the marriage market is a good appearance, whereas the corresponding quality for men is the ability to earn a living (Klasen and Pieters 2015). For the younger cohort, a recent study found that economic potential, trustworthiness, and intelligence of the prospective partner are increasingly more valued than other traits, and this is true for both men and women (Prakash and Singh 2014). Even so, in terms of education, women are still expected to marry those with higher education than themselves, and women who have more education than their husbands are seen as gender-deviant and may face consequent repercussions. For example, a recent study using a nationally representative sample of Indian women found that women with higher education who earn more than their spouses have the highest likelihood of experiencing intimate partner violence: they are thought to threaten men’s dominant status in the household, and men respond by using violence (Weitzman 2014).

Educational expansion in India has occurred through the establishment of new and often privately run schools, colleges, and distance education. Given the little control over the quality of education delivered by private (and often public) institutions in India, independent assessments of learning outcomes document the declining quality of skills over time (ASER Centre 2013). One of the major issues complicating research on educational assortative mating over time concerns how the implications of educational expansion for mate selection vary across social contexts and social groups. For example, although women are more likely to be involved in higher education than before, they are still more concentrated in fields that have traditionally been considered feminine, such as humanities and social sciences. Men are more likely to be in science, technology, engineering, and mathematics (STEM) fields, which often have to higher economic returns in the labor market (England and Li 2006). Similarly, in India, the education system is highly gendered, with women being more likely to be tracked into arts (humanities and social sciences) and men being more likely to be tracked into STEM and commerce. Hence, education may not have the same signaling value for men and women.

Our calculation from the India Human Development Survey (IHDS) documents that, among men aged 20–30, 11% had a college degree in 2004–2005, compared with 14% by 2011–2012. For women, the commensurate increase was sharper, from 8% to 12%. However, most of this growth occurred in the arts fields. For those with a college degree, arts degrees recorded a modest decrease for men but a significant increase for women: in 2011–2012, 40% of men with a college degree majored in the arts, compared with as many as 70% of college graduate women. Employment opportunities for graduates outside STEM and commerce have consistently declined over time in India, even for men. Although educational attainment has grown sharply, the proportion of jobs in the formal sector has expanded very slowly, impacting job opportunities for college graduates (Papola 2012).

Salaried jobs in government or the formal sector—as opposed to casual labor or self-employment in petty business or farming—hold the key to economic prosperity in India (Desai et al. 2010). Our calculations from the IHDS show that for men with arts degrees, 47% of the cohort aged 40–49 were able to find salaried jobs, but this figure dropped to 40% for men aged 30–39. However, for college graduates in STEM and commerce, the percentage finding salaried jobs held steady at 55%. The situation was even worse for women, with only 30% of non–STEM/commerce major women being involved in any salaried jobs (either part-time or full-time jobs). This percentage was slightly higher for STEM–major women—38% were able to find salaried jobs—but it still lagged behind that of men.

If the lack of employment opportunity, particularly for women with arts degrees, plays an important role in shaping women’s market options, changes in assortative mating may have less to do with the global forces outlined here than with the changing salience of educational attainment in India.

## Research Motivations: Emergence of Educational Hypogamy in India

**Fig. 1:**
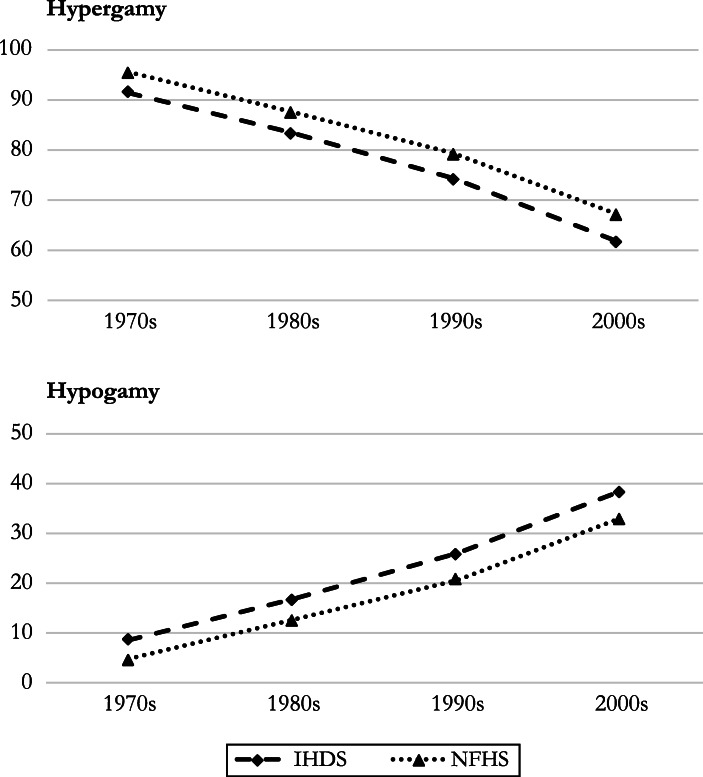
Percentage of educational hypergamy (H > W) and hypogamy (H < W) given heterogamy (H ≠ W) by marriage cohort and data source. H = husband’s education; W = wife’s education. *Sources:* 2011–2012 India Human Development Survey (IHDS) and 2014–2015 National Family Health Survey (NFHS).

Consistent with the global trend of educational assortative mating, India has also experienced a dramatic decrease in hypergamous marriages and a considerable increase in hypogamous marriages. Figure 1 shows trends in the percentage of hypergamous and hypogamous married couples among heterogamous couples (who have different levels of education). We show results from both the IHDS and National Family Health Survey (NFHS) for the purpose of cross-validation, and despite slight differences between two data sources, the trends of hypergamy and hypogamy are exactly the same. During the last four decades, the percentage of husbands having more education than wives among couples with different levels of education declined from more than 90% to around 60%, and the percentage of women marrying men with lower education increased from less than 10% to more than 30%.

**Fig. 2:**
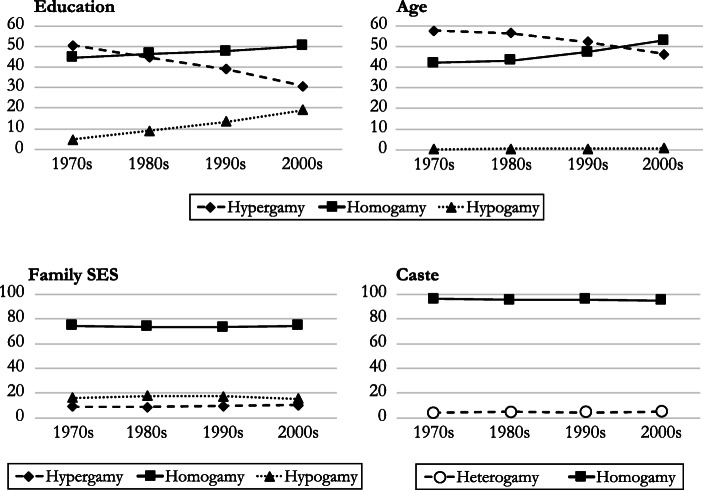
Assortative mating by education, age, family SES, and caste from 1970 to 2012. *Source*: 2011–2012 India Human Development (IHDS).

However, the incidence of crossing other marriage boundaries exhibited little change during the same period. Besides educational assortative mating, Fig. 2 also shows changes in the assortative mating patterns by age, family socioeconomic status (SES), and caste for Indian marriages between 1970 and 2012. The phenomenon of a woman marrying a man of the same age or higher age is still prevalent, and wives are older than husbands in less than 1% of the marriages. With regard to family background, most couples are from families with similar SES, and intercaste marriage is rare. The greatest change in mate selection appears to be in terms of the increase in educational hypogamy. The question of why the boundary of education is less salient than others, therefore, is critical to our understanding of educational assortative mating in a very rigid marriage market.

These figures provide the motivation for this study as we systematically examine the trends and patterns of educational assortative mating in India using a nationally representative data set. We first examine the role of general educational expansion in accounting for changes in assortative mating patterns. Next, in our analyses of the determinants of educational hypogamy, we explore the role of the family SES background; the local marriage pool; and other cultural/normative constraints, such as caste, religion, and other marriage traditions. Considering the persistence of gender hierarchy, the preference for hypergamy and traditional marriage practices, and low economic returns to women’s education, we expect that the role of women’s education in the Indian marriage market may be exhibited in different ways from other contexts.

We formulate our hypotheses along two major lines. First, following the status exchange theory, we expect a trade-off between education status and economic standing. Women with higher levels of education but from a natal family with lower economic standing could have a higher likelihood of an educational hypogamy: *marrying up* economically while *marrying down* educationally. Similarly, we expect that women in fields of study with lower economic returns are more likely to be in a relationship characterized by educational hypogamy. Second, we expect the salience of other social and cultural constraints to exert pressure on the penetration of education as a social boundary in assortative mating. In states that have a relative shortage of eligible men with higher educational levels in the marriage pool, we expect the odds of hypogamy to be higher. Similarly, marrying within the family (consanguineous marriage) could increase one’s odds of educational hypogamy when the partner pool is small to begin with.

## Data

We use data from the IHDS, an ongoing longitudinal study documenting changes in the daily lives of Indian households (Desai et al. 2015). The IHDS is a nationally representative survey of 41,554 households in the first wave (2004–2005) and 42,152 households in the second wave (2011–2012), including original households, households that split from original households, and a replacement sample of additional households. This study uses data from the 2011–2012 wave of this survey (IHDS-II) because some key variables measuring marriage practices are available only in this wave. During the survey, marriage-related retrospective questions were asked to ever-married women in the selected households, which makes IHDS-II especially appropriate for studying diverse assortative mating patterns in India.^1^ We restrict our sample to women’s first-marriage history to ensure that the trends in assortative mating over time are not affected by marriage order. Given that remarriage is rare in India, it has only a minor impact on our sample.^2^ After the exclusion of observations with missing values on analytical variables and 51 women who were first married before 1970, the analytical sample contains 28,311 women with detailed information on their first marriage.

To supplement this analysis, we also draw state-level data from the National Sample Surveys (NSS) of India to help us address our research questions. Using the NSS (Rounds 38, 43, 50, 55, 61, and 66), we calculate changes in the educational distribution of marriage-eligible men (aged 20–29 years) and women (aged 15–24 years) in each state of India at six time points: 1983–1984 (Round 38), 1987–1988 (Round 43), 1993–1994 (Round 50), 1999–2000 (Round 55), 2004–2005 (Round 61), and 2009–2010 (Round 66). We combine Rounds 38 and 43 to calculate the state-level pool of eligible persons for the marriage market in the 1970s and 1980s, Rounds 50 and 55 for that in the 1990s, and Rounds 61 and 66 for that in the 2000s. Serving the same function as the Current Population Surveys (CPS) in the United States, the NSS are the only surveys providing comparable information over a long period for India, with a sample of more than 100,000 households and about 500,000 individuals (Desai and Kulkarni 2008). The NSS thus allows for a large sample from which to calculate the state-level educational distribution for the pool of eligible mates.

## Variables

The topic of interest—educational assortative mating—is measured by comparing the highest educational attainment of women and their husbands. We measure the highest educational level attained by the respondents at the time of the interview, in the following five categories: (1) no formal education, (2) primary school, (3) secondary school, (4) higher secondary school and some college, and (5) college degree or higher. Indian women rarely pursue further formal education after getting married, especially given that patrilocal extended families are still prevalent in India (Allendorf 2013). Thus, our use of educational attainment at the time of survey is a good proxy for education at the time of marriage. This measure has also been widely used in studying educational assortative mating in other non-Western countries (Gullickson and Torche 2014; Qian and Qian 2014). To examine trends in educational assortative mating, ever-married women in this sample are categorized into the following three marriage cohorts based on the time of their respective first marriages: the 1970s–1980s, the 1990s, and the 2000s.^3^

To study how economic returns to women’s education are associated with assortative mating, in our multivariate analyses, in addition to the highest educational attainment of women, we further divide college-educated women into two groups based on their school fields.^4^ Considering different labor market outcomes across college majors in the Indian context, we further divide college education into two groups based on fields: one majoring in arts^5^ and the other related to STEM and commerce.^6^

IHDS-II collected data on a variety of marriage-related characteristics from ever-married women. One key independent variable of this study is the comparison of family economic status between the families of the brides and grooms, measured on the basis of the wives’ reports of how their husbands’ family SES compared with their own at the time of marriage. The response options are as follows: (1) same, (2) natal family better off, or (3) natal family worse off. Because our focus of the study is to test whether women’s marrying down in education is associated with marrying up in the family SES, we combine the first two groups to make a dichotomous variable measuring whether the SES of the husbands’ families is better than that of the wives’ families.^7^

We also include women’s age at marriage and spousal age differences in our analysis. Women’s responses to the question about whether they belong to the same caste as their husbands are used to define intercaste marriage. Finally, we take into account the arranged marriage and consanguineous marriage. We code the marriages decided by parents or relatives as arranged marriages, by women themselves as self-decided marriages, and by both women and parent/relative together as jointly decided marriages. Consanguineous marriage is measured by the question, “Are you related to your husband by blood?”; a response of “yes” is categorized as a consanguineous marriage.

Because caste and religion are the major axes of stratification in the Indian context, we further divide our sample of women based on their identification of caste and religion. Following a previous study using the same data set (Thorat et al. 2017), we use a fourfold classification of caste: Other Backward Castes (OBC), Dalits (Scheduled Castes), Adivasis (Scheduled Tribes), and all others (mostly Forward Castes). As for religion, women were divided into four groups: Hindus; Muslims; Sikhs and Christians; and all other religions, which include Buddhists, Jains, others, and no religious affiliation.

To control for educational expansion and the availability of men and women with various levels of education, we use the NSS data set to construct several state-level variables measuring the marriage pool of each state/union territory. We calculate the relative proportion of men (aged 20–29 years) to women (aged 15–24 years) in each educational category for each of the 33 states/union territories in our sample.

## Analytic Strategies

We estimate several statistical models based on different research questions. We start with a series of log-linear models to analyze contingency tables to describe changes in patterns of educational assortative mating in the Indian marriage market. Log-linear models are the gold standard for most statistical analyses of assortative mating (Blossfeld 2009). These models are preferred for estimating associations between the spouses’ characteristics while controlling for husband–wife differences in the marginal distributions of these characteristics (Kalmijn 2010). Then, to incorporate our covariates and examine whether different educational matching patterns are associated with exchange for family economic status as well as other marriage practices, we estimate a series of multilevel logistic regression models that predict hypogamy as a function of the couples’ characteristics as well as the characteristics of state-level marriage pool (Hou and Myles 2013; Tsay and Wu 2006).

First, we produce a contingency table with 75 cells (5 × 5 × 3) by cross-tabulating the husbands’ education with the wives’ education for the three marriage cohorts. Each cell represents the number of first marriages between individuals with specific levels of education within a given marriage cohort. The diagonal cells in the contingency table represent homogamous marriages, and the off-diagonal cells represent hypergamous and hypogamous marriages. We start with a baseline model, assuming no changes in the couples’ education across cohorts. Then, to examine how education expansion is associated with changing matching patterns, we develop the following model, allowing two-way interactions among the couples’ education by marriage cohorts:

1 $$ \log {F}_{ij k}={\upbeta}_0+{\upbeta}_i^H+{\upbeta}_j^W+{\upbeta}_k^C+{\upbeta}_{ik}^{HC}+{\upbeta}_{jk}^{WC}+{\upbeta}_{ij}^{HW}, $$

where *H* is the husband’s education (*i* = 1–5), *W* is the wife’s education (*j* = 1–5), and *C* (*k* = 1–3) is marriage cohort. Thus, *F*_*ijk*_ is the expected number of marriages between husbands with education *i* and wives with education *j* from marriage cohort *k*. This model captures changes in the distribution of the education of the husband and wife by the marriage cohort (${\upbeta}_{ik}^{HC}$ and ${\upbeta}_{jk}^{WC}$) and also controls for the time-invariant association between the spouses’ educational attainment (${\upbeta}_{ij}^{HW}$).

To model the trends for different educational matching patterns, we add three parameters—indicating homogamy, hypergamy, and hypogamy—to the baseline model separately. Each parameter is a 5 × 5 matrix with 1 in the diagonal or one side of off-diagonal cells and 0 elsewhere. The updated equations are as follows:

2 $$ \log {F}_{ij k}={\upbeta}_0+{\upbeta}_i^H+{\upbeta}_j^W+{\upbeta}_k^C+{\upbeta}_{ik}^{HC}+{\upbeta}_{jk}^{WC}+{\upbeta}_{ij}^{HW}+{\upbeta}_o^{OC}\left(\mathrm{or}\ {\upbeta}_p^{PC}\mathrm{or}\ {\upbeta}_y^{YC}\right), $$

where ${\upbeta}_o^{OC}$, ${\upbeta}_p^{PC}$, and ${\upbeta}_y^{YC}$ allow variation in the homogamy, hypergamy, and hypogamy parameters, respectively, by marriage cohort.

Although log-linear models help to establish the association between husbands’ and wives’ education with marginal distributions fitted, they do not reveal what specifically could associate with assortative mating (Hu and Qian 2019). As a second step, we employ a series of multilevel logistic regression models to predict women’s hypogamy in education. Because only women who have attained at least primary level education have the option to marry down, we limit our sample to those who have completed at least primary school. We compute stepwise logistic models adding each key independent predictor (cultural constraints on mate selection, comparison of the SES of the bride’s family with that of the groom’s, and educational category) to a baseline model that regresses hypogamy on marriage cohort and controls. We also include some interaction terms among the key predictors to examine whether they jointly influence the probability of hypogamy. To account for the constraints faced by women (Level 1) in mate selection from the availability of qualified men in the local marriage market (Level 2), we estimate the two-level random intercept logistic regression models, including state-level variables measuring the relative proportion of men (aged 20–29) to women (aged 15–24) in each education category. Multilevel modeling corrects for the biases in parameter estimates resulting from clustering and thus reports robust standard errors (Guo and Zhao 2000). All multilevel analyses were fitted using maximum likelihood estimation by using the *xtmelogit* command in Stata 15.

## Results

Table 1 shows the distribution of education for husbands and wives by marriage cohorts. Consistent with the global trend of educational expansion, both husbands and wives experienced a dramatic increase in their respective levels of educational attainment. Nearly one-half of the wives and one-quarter of the husbands never received any formal education among those married in the 1970s–1980s, but most of the husbands and wives married in the 2000s had at least five years of education. At the same time, gender differences in higher education also dropped monotonically. In the 1970–1980s marriage cohort, husbands were three times more likely to have a college degree than their wives; in the 2000s cohort, there were only small differences in degree attainment (13.9% for men vs. 11.1% for women).

**Table 1:** Percentage distribution of education for husbands and wives by marriage cohort

	Husband (%)					
Wife	None	Primary	Secondary	Higher Secondary	College Graduate	Total
1970–1980s						
None	20.3	11.6	12.1	1.2	0.7	45.9
Primary	1.9	5.7	10.0	1.0	0.7	19.3
Secondary	1.0	2.9	16.5	3.4	3.9	27.6
Higher Secondary	0.0	0.1	1.0	0.8	1.5	3.4
College Graduate	0.0	0.0	0.6	0.4	2.8	3.8
Total	23.1	20.4	40.1	6.8	9.6	100.0
*N*						8,661
1990s						
None	12.0	6.3	8.8	1.2	0.3	28.6
Primary	2.1	4.8	8.4	0.9	0.4	16.5
Secondary	1.8	4.5	24.3	5.7	4.1	40.4
Higher Secondary	0.1	0.2	2.6	1.9	2.8	7.6
College Graduate	0.0	0.0	1.1	1.2	4.5	6.9
Total	16.0	15.9	45.0	10.9	12.2	100.0
*N*						8,520
2000s						
None	6.2	4.2	5.6	0.5	0.1	16.6
Primary	1.7	3.6	6.4	0.7	0.2	12.6
Secondary	2.0	5.1	29.6	6.2	3.5	46.5
Higher Secondary	0.1	0.3	5.4	4.1	3.3	13.2
College Graduate	0.0	0.1	2.1	2.3	6.6	11.1
Total	10.1	13.2	49.0	13.8	13.9	100.0
*N*						11,130

To further examine how educational expansion is associated with different matching patterns, we apply log-linear models to explore educational assortative mating patterns net of differences in the marginal distributions of spouses’ educational attainment. Table 2 reports the goodness-of-fit statistics for each log-linear model examined in this study. We use the likelihood ratio test to compare models including parameters indicating different matching patterns with Model 2 as specified in Eq. (1). The likelihood ratio tests indicate that the model fit increases at the .05 level of significance as the homogamy parameter (Model 3) and the hypogamy parameter (Model 5) are added to Model 2 separately. However, adding the hypergamy parameter (Model 4) does not significantly improve the model fit relative to Model 2. Because Model 2 accounts for education expansion for both husbands and wives, we conclude that changes in the educational distributions of both wives and husbands explain most of the declines in marriage hypergamy. On the other hand, the incidence of women marrying below their educational status has increased significantly even after controlling for increases in women’s educational attainment.

**Table 2:** Log-linear models of the association between husband’s and wife’s educational attainment: India, 1970–2012

Model	Description	Specifications	*df*	Deviance	*p* (vs. Model 2)	BIC
1	Baseline	[HW] [C]	49	3,258	––	2,756
2	Model 1 + H × C + W × C	[HW] [HC] [WC]	33	71	––	–267
3	Model 2 + C × Homogamy	[HW] [HC] [WC] [OC]	31	64	.03	–254
4	Model 2 + C × Hypergamy	[HW] [HC] [WC] [PC]	31	71	.88	–247
5	Model 2 + C × Hypogamy	[HW] [HC] [WC] [YC]	31	53	.00	–265

*Notes*: H = Husband’s education, W = Wife’s education, C = Cohort, O = Homogamy, P = Hypergamy, and Y = Hypogamy. BIC = Bayesian information criterion.

Based on the results from Models 3, 4, and 5 in Table 2, we display parameters on homogamy, hypergamy, and hypogamy in Table 3. The results suggest that educational expansion explains most of the trends in hypergamy and homogamy. The odds of homogamy declined slightly—by 9% in the 1990s and by 8% in the 2000s—compared with those in the 1970s–1980s. With regard to hypergamy, though, its proportion in all marriages declined dramatically (from 46% to 30%); our results from log-linear models show that the decline was mostly driven by changes in the marginal distribution of the education of the husband and that of the wife. Net of these distributional changes, education hypergamy in India actually experienced no statistically significant changes during the last four decades. However, the incidence of hypogamy increased significantly during the same period. Consistent with the results shown in descriptive statistics, those from log-linear analysis reveal that net of changes in educational distribution, the odds of women marrying below their educational status are 27% and 28% higher, respectively, among marriages in the 1990s and the 2000s compared with those in the 1970s–1980s.^8^

**Table 3:** Select parameter estimates for educational assortative marriage from Models 3–5 in Table 2 (log-linear models)

	β	Exp(β)
Model 3: Educational Homogamy (ref. = 1970s–1980s)		
1990s	–0.091*	0.913
2000s	–0.083*	0.920
Model 4: Educational Hypergamy (ref. = 1970s–1980s)		
1990s	0.024	1.024
2000s	–0.002	0.998
Model 5: Educational Hypogamy (ref. = 1970s–1980s)		
1990s	0.236***	1.266
2000s	0.243***	1.275

*p < .05; ***p < .001

These findings differ substantially from the trends observed in other social contexts. For example, studies in diverse contexts such as in the United States and China have shown that educational expansion is often followed by a substantial decline in hypergamy and an increase in homogamy even after the distribution is controlled for (Esteve et al. 2016; Han 2010; Schwartz and Mare 2005). The incidence of hypogamy outnumbers that of hypergamy in the United States and many European countries (Grow and Van Bavel 2015; Van Bavel et al. 2018) only after the reversal of the gender education gap. Our findings, however, reveal a different picture of trends in education assortative mating, showing a net increase only in hypogamy and little change in both hypergamy and homogamy.

To further examine the social processes associated with the phenomenon of Indian women marrying below their educational status, we estimate a series of multilevel logistic regression models predicting hypogamy. Table 4 presents the basic summary statistics for variables included in regression models by homogamous/hypergamous and hypogamous marriages separately. The differences between these two types of marriages are statistically significant in most variables except for marriage arrangement, spousal age differences, and intercaste marriage.

**Table 4:** Descriptive characteristics of women by marriage type

Variables	Total	Homogamy/Hypergamy	Hypogamy	
Marriage Cohort (%)				*
1970–1980s	23.4	24.9	17.2	
1990s	30.3	30.6	29.1	
2000s	46.3	44.5	53.7	
Marriage Arrangements (%)				ns^a^
Arranged	68.4	68.7	66.9	
Self-decided	6.4	6.3	7.0	
Jointly decided	25.2	25.0	26.1	
Consanguineous Marriage (%)	6.7	6.2	8.7	*
Age at Marriage	19.2	19.1	19.6	*
	(0.0)	(0.0)	(0.0)	
Age Differences (Husband-Wife)	5.3	5.3	5.3	ns^a^
	(0.0)	(0.0)	(0.1)	
Intercaste Marriage (%)	5.2	5.2	5.3	ns^a^
Husband’s Family SES Better (%)	9.4	9.7	8.3	*
Education Category				*
Primary/secondary	77.2	82.6	55.5	
Higher secondary	12.1	8.9	24.8	
College (arts)	7.1	5.6	13.2	
College (STEM/commerce)	3.6	2.9	6.5	
Caste (%)				*
Forward castes and other religions	38.0	39.1	33.6	
Other backward castes (OBC)	38.8	38.0	42.1	
Dalits	17.7	17.5	18.3	
Adivasis	5.5	5.4	6.0	
Religion (%)				*
Hindus	82.0	83.1	77.5	
Muslims	10.0	9.3	13.2	
Sikhs and Christians	6.4	6.0	7.7	
Others	1.6	1.6	1.6	
*N*	19,909	15,976	3,933	

^a^Not significant.

*p < .05 (signifies significant differences between homogamy/hypergamy and hypogamy based on chi-square test or *t* test)

Table 5 presents multilevel logistic regression models predicting women’s marrying below their educational status. Model 1 is the baseline model including only marriage cohorts and control variables, and Model 2 adds state-level variables of educational marriage pool. Results show that state-level variables measuring the local marriage market are closely associated with women’s martial choice. Women are less likely to be involved in hypogamous marriages when there is an abundance of eligible men in their local marriage market. Furthermore, coefficients of marriage cohorts have declined considerably from Model 1 to Model 2, suggesting that localized educational expansion may help explain a significant proportion of changes in educational assortative mating.

**Table 5:** Multilevel logistic regression models predicting educational hypogamy

Variables	Model 1	Model 2	Model 3	Model 4	Model 5
Individual-Level Variables					
Marriage cohort (ref. = 1970–1980s)					
1990s	0.316***	0.259***	0.242***	0.117	0.122
	(0.053)	(0.063)	(0.063)	(0.066)	(0.073)
2000s	0.552***	0.406***	0.366***	0.134	0.038
	(0.049)	(0.080)	(0.080)	(0.087)	(0.092)
Marriage arrangement (ref. = arranged)					
Self-decided			0.069	–0.030	–0.033
			(0.077)	(0.081)	(0.081)
Jointly decided			0.024	–0.044	–0.046
			(0.044)	(0.046)	(0.046)
Consanguineous marriage			0.185**	0.203**	0.201**
			(0.070)	(0.073)	(0.073)
Age at marriage			0.021***	–0.049***	–0.049***
			(0.006)	(0.006)	(0.006)
Age differences			–0.006	–0.010	–0.010
			(0.006)	(0.006)	(0.006)
Intercaste marriage			–0.034	–0.045	–0.046
			(0.083)	(0.087)	(0.087)
Husband’s family SES (better = 1)			–0.124	–0.126	–0.182*
			(0.065)	(0.068)	(0.086)
Education category (ref. = primary/secondary)					
Higher secondary				1.610***	1.518***
				(0.052)	(0.137)
College (arts)				1.652***	1.142***
				(0.069)	(0.173)
College (STEM/commerce)				1.451***	1.529***
				(0.090)	(0.224)
Husband’s family SES × education category					
Husband better × higher secondary					–0.067
					(0.171)
Husband better × college (arts)					0.557**
					(0.227)
Husband better × college (STEM/commerce)					0.249
					(0.299)
Education category × marriage cohort					
Higher secondary × 1990s					–0.066
					(0.164)
Higher secondary × 2000s					0.213
					(0.150)
College (arts) × 1990s					0.265
					(0.207)
College (arts) × 2000s					0.678***
					(0.188)
College (STEM/commerce) × 1990s					–0.057
					(0.270)
College (STEM/commerce) × 2000s					–0.123
					(0.247)
Caste (ref. = forward castes and other religions)					
Other backward castes (OBC)	0.127**	0.130**	0.152***	0.330***	0.320***
	(0.045)	(0.045)	(0.045)	(0.047)	(0.047)
Dalits	0.139**	0.142**	0.173**	0.485***	0.480***
	(0.054)	(0.054)	(0.055)	(0.058)	(0.058)
Adivasis	0.223**	0.234**	0.248**	0.528***	0.530***
	(0.085)	(0.084)	(0.085)	(0.089)	(0.089)
Religion (ref. = Hindus)					
Muslims	0.349***	0.351***	0.363***	0.575***	0.577***
	(0.058)	(0.058)	(0.059)	(0.062)	(0.062)
Sikhs and Christians	0.191*	0.186*	0.158*	0.215*	0.210*
	(0.081)	(0.080)	(0.081)	(0.084)	(0.085)
Others	0.191	0.181	0.170	0.115	0.107
	(0.149)	(0.149)	(0.149)	(0.155)	(0.155)
State-Level Variables					
Relative proportion of men (aged 20–29) to women (aged 15–24) in:					
Primary education		0.337**	0.330**	0.138	0.124
		(0.116)	(0.115)	(0.124)	(0.124)
Secondary education		–0.068	–0.059	–0.077	–0.034
		(0.126)	(0.125)	(0.135)	(0.135)
Higher secondary education		–0.236*	–0.217*	–0.261*	–0.278*
		(0.103)	(0.101)	(0.112)	(0.113)
College education		–0.001	–0.001	–0.001	–0.001
		(0.001)	(0.000)	(0.001)	(0.001)
Constant	–1.968***	–1.866***	–2.303***	–1.125***	–1.090***
	(0.069)	(0.180)	(0.233)	(0.254)	(0.255)
State-Level Variance	0.055	0.037	0.033	0.057	0.057
	(0.018)	(0.013)	(0.012)	(0.021)	(0.021)
Intraclass Correlation	0.017	0.011	0.010	0.017	0.017
Log-Likelihood	–9,687	–9,679	–9,665	–9,024	–9,009
*N*	19,909	19,909	19,909	19,909	19,909

*p < .05; **p < .01; ***p < .001 (two-tailed tests)

We add a series of variables measuring cultural norms on marriage practices in Model 3 to test whether these factors are associated with hypogamous marriages. The results indicate that consanguineous marriages (e.g., marriages among cousins) are 20% more likely to be hypogamous than marriages not related by blood (exp(0.185) = 1.203), which can be suggestive of a strong cultural constraint on spousal selection in certain parts of India.

Model 4 of Table 5 further includes the variable indicating fields of education. Apart from the primary and secondary levels for the omitted category, the other categories are higher secondary, college degrees in arts, and college degrees in STEM/commerce. The results show that women with higher secondary education and beyond have a higher likelihood of entering into a hypogamous marriage than those with secondary education and below. Specifically, the odds of hypogamy are five times as likely among women with higher secondary education (exp(1.610) = 5.002) and college education majoring in arts (exp(1.652) = 5.217) than among women with secondary level education and below, and this is slightly lower (four times) among women with STEM/commerce college degrees (exp(1.451) = 4.267).

**Fig. 3:**
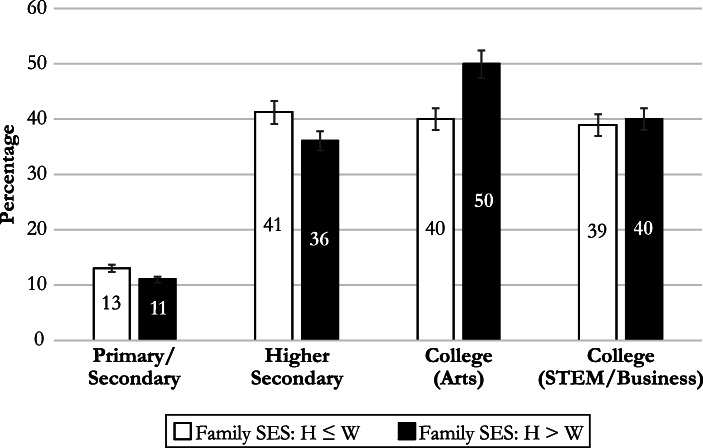
Predicted probabilities of educational hypogamy (H < W) by family SES and educational category (with 95% confidence intervals). Results are based on Model 5 of Table 5. The model also includes marriage cohort, a variety of marriage-related characteristics, caste, religion, and state-level variables measuring the educational distribution for the pool of eligible mates.

In Model 5, we include two interaction terms: one between the education category and the SES of the husband’s family, and the other between the education category and the marriage cohort. Model 5 tests whether the exchange of education for the family SES varies across the education category. We find that although the main effect of the family SES is negatively associated with hypogamy, it becomes positive for women with college degrees in arts. To aid interpretation, we show the predicted probabilities of hypogamy for the exchange of the family SES by the educational category in Fig. 3. We find that among women with arts degrees, they often seem to trade economic status for education: predicted probability of marrying a man with lower education is the highest among women with arts degrees (50%).

**Fig. 4:**
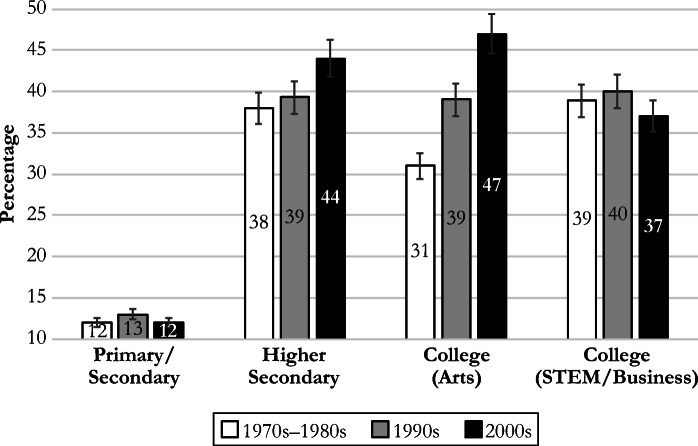
Predicted probabilities of educational hypogamy (H < W) by marriage cohort and educational category (with 95% confidence intervals). Results are based on Model 5 of Table 5. The model also includes a variety of marriage-related characteristics, caste, religion, and state-level variables measuring the educational distribution for the pool of eligible mates.

The results from Model 5 further show that the disadvantage of women with college degrees in arts grew over time. As shown in Fig. 4, the probability of hypogamy among college-educated women majoring in arts was only 31% for the 1970s–1980s marriage cohort. However, it increased to 39% for the 1990s cohort and to 47% among the marriage cohort of the 2000s, with all other variables in the model held constant. The trend of hypogamy among the other three groups shows no statistically significant changes from the 1970s–1980s to the 2000s. Thus, much of the increase in hypogamy seems to lie among women with arts degrees. Considering the segmented educational system by gender through which women are more likely to be tracked into arts but men are more likely to be tracked into STEM/commerce, and substantial gender asymmetry in the labor market payoffs of education, the results of our study suggest that educational expansion with a focus on arts degrees without concomitant employment expansion may dilute the value of education in the marriage market in the context of serious gender hierarchies.

## Discussion and Conclusion

The expansion of women’s education around the world has led to dramatic changes in the composition of the marriage market and patterns of assortative mating. Our analyses of recent marriage cohorts in India using the IHDS (2011–2012) demonstrate consistency with the global trend in general while also showing notable differentiation. Consistent with the global pattern, the expansion of women’s education has significantly reduced the incidence of hypergamy in India. Accompanied by a steady decline in hypergamy in three adjacent marriage cohorts (the 1970s–1980s, 1990s, and the 2000s), we observe a notable increase in hypogamy over time. Whereas hypogamy has been increasing for Western countries with a reverse gender gap in education, an increase in homogamy rather than hypogamy has been more commonly observed in the non-Western countries (Han 2010; Qian and Qian 2014; Smits and Park 2009).

What makes the case of India stand out? Does such an increase in the incidence of educational hypogamy suggest that marriage has become more egalitarian and flexible in India? Our answer is a definitive no. Despite a considerable proportion of Indian women marrying men with lower education than themselves, other boundaries typical of a patriarchal marriage regime remain remarkably rigid. For example, women marrying younger men are still rare, as is intercaste marriage or marrying men from a lower economic standing. A closer examination of the IHDS data leads us to a much more nuanced understanding of educational assortative mating patterns in India. First, our log-linear analyses show that the increase in educational hypogamy for recent marriage cohorts is expectedly a function of expansion in education: levels of educational attainment improve significantly for men and women, accompanied by a reduction in the gender gap in education over time. However, that is not the whole story. Although improvement in women’s education makes it possible for women to marry down (in terms of education level), our follow-up multilevel logistic regression models reveal several factors contributing to educational hypogamy. First, for women with a higher level of education who marry down in terms of educational matching, they are more likely to come from natal families with a lower economic status than their husbands’ family. This is consistent with the previous empirical evidence on the status exchange theory showing a pattern of intermarriage across social origin and education boundaries (Qian and Qian 2017; Schwartz et al. 2016).

Thus, such a trade-off between one’s education and natal family’s economic standing suggests that economic resources could be a more important criterion in mate selection than education credentials. Our additional analyses provide further evidence for this argument. We find that women majoring in fields that are typically associated with worse labor market outcomes in the Indian context (e.g., humanity and social sciences instead of STEM) are increasingly more likely to be involved in hypogamous marriages. As observed in many transitional economies, expansion of education is often uneven, with an increasing diversity in its returns manifested in the labor market outcomes, and this may be particularly true for women (World Bank 2018). As a whole, female labor force participation in India remains low, even for those with college degrees (Chatterjee et al. 2018; Klasen and Pieters 2015). The failure to translate educational advantages into economic gains means that women who marry down in terms of educational attainment are still very likely to earn less than their husbands. Earning or economic standing could be a far more rigid boundary than education to cross.

Other than SES, there are other salient social boundaries in place that are unique to the Indian context. Our analyses demonstrate that the rigidity of these boundaries could increase the likelihood of educational hypogamy. Our evidence is indirect but plausible. For example, our results suggest that consanguineous marriage is positively associated with hypogamy. In certain parts of India where the norm to marry within the family is strong and the eligible pool is obviously small, marrying men with lower education could be more of a necessity. Similarly, our multilevel analysis measuring the state-level marriage pool tells a story that is suggestive of another cultural constraint in operation. We find that the size of the eligible pool of men—that is, those with higher secondary education—is negatively associated with the odds of educational hypogamy. In other words, women are much less likely to marry below their educational status when there is an abundance of eligible men. Conversely, in absence of a large eligible pool of men, given that universal marriage is the norm, marrying down in education could be a woman’s only option when the choice of not getting married simply does not exist. All in all, fluidity in educational assortative mating in India could precisely be an adaptive outcome when institutional norms and other cultural and social boundaries are salient and relatively impenetrable.

Certain limitations of this study are worth noting. First, we use college fields (STEM/commerce vs. arts) as a proxy of economic returns to education. In other explorative analyses, we conducted the same analysis using the level of English proficiency as a proxy, and results were generally similar. However, we acknowledge that individuals’ choice of college fields is highly selective and varies significantly by their social backgrounds. Also, despite numerous studies on educational assortative mating all around the world, very few of them have taken the field of education into account. We suggest that future studies on educational assortative mating take more nuanced measures of education into consideration.

Second, to account for the constraints faced by women in mate selection from the availability of qualified men in the local marriage market, we include state-level variables measuring the marriage pool of each state. However, states might be a geographical unit that is too broad to effectively measure the local marriage market in India. Unfortunately, detailed information on marriage pools at a lower geographic level from the 1970s to 2000s is lacking. Finally, although IHDS is an ongoing longitudinal survey, some important variables on marriage assortative mating are available only from the 2011–2012 wave, and thus this study is cross-sectional in nature. With IHDS-III expected to be available in upcoming years, future studies can make use of longitudinal data to rule out potential selection issues.

Despite these limitations, our study is among the first to provide empirical evidence on assortative mating patterns in a social context of arranged marriages and hierarchical gender regimes. In India’s unique social and economic panorama, the rise in educational hypogamy does not suggest a breakdown of gender barriers or a promising shift in gender norms. On the contrary, it could indeed be indicative of other forms of deep-running roots of social and gender stratification in the Indian society. Undoubtedly, expansion in women’s education should be considered real progress, but it is a first step toward gender equality rather than a sufficient condition for it. The stalled gender revolution in the Western countries has already provided us with cautionary tales, where the reversed gender gap in education has certainly not led to pay equity in the labor market. Thus, the emergence of educational hypogamy in India does not herald a shift toward gender egalitarianism but is rather indicative of other stronger structural barriers in the long road to achieving women’s empowerment.

## Data Availability

The data that support the findings of this study are available in the Data Sharing for Demographic Research at https://www.icpsr.umich.edu/web/DSDR/studies/36151, reference number ICPSR 36151.

## Footnotes

1 The National Family Health Survey (NFHS), the Indian equivalent of the Demographic and Health Survey (DHS), also provides information on the educational attainments of couples and the year of marriage. This data set is hence available for studying educational assortative mating over time. As shown in Fig. 1, the results from the IHDS and the NFHS are largely similar.

2 Less than 1% of women’s first marriage ended in divorce or separation in this sample.

3 In an earlier analysis, we separated the marriage cohort of the 1970s from that of the 1980s and did not find any changes in the results regarding the trend of educational assortative mating and its associates. We combine these two cohorts because the sample size of women who married in the 1970s is relatively small (7.8%), and combining it with the marriage cohort of the 1980s (22.8%) can help enhance statistical power.

4 In an earlier analysis, we also used English proficiency as a proxy, and results were largely similar.

5 In the Indian educational system, arts degrees consist of humanities and social sciences.

6 In our sample, about 1% of women major in other fields, such as law and design, and results with and without them are exactly the same. Because our preliminary analysis showed their characteristics to be similar to those in STEM and commerce, we include them in that category.

7 In an exploratory analysis, the comparison of family economic status between the families of the brides and grooms was also measured by the difference between the educational attainment of the father of the respondent and that of her husband. The results were generally similar to the findings of this study.

8 Additional analyses, with the reference group being the marriage cohort of the 1990s, show no statistically significant differences between the 1990s and 2000s.
